# Reciprocal transplantation of the heterotrophic coral *Tubastraea coccinea* (Scleractinia: Dendrophylliidae) between distinct habitats did not alter its venom toxin composition

**DOI:** 10.1002/ece3.5959

**Published:** 2020-02-06

**Authors:** Marcelo V. Kitahara, Adrian Jaimes‐Becerra, Edgar Gamero‐Mora, Gabriel Padilla, Liam B. Doonan, Malcolm Ward, Antonio C. Marques, André C. Morandini, Paul F. Long

**Affiliations:** ^1^ Departamento de Ciências do Mar Universidade Federal de São Paulo Santos Brazil; ^2^ Centro de Biologia Marinha (CEBIMar) Universidade de São Paulo São Sebastião Brazil; ^3^ Departamento de Zoologia Instituto de Biociências Universidade de São Paulo São Paulo Brazil; ^4^ Departmento de Microbiologia Instituto de Ciências Biomédicas Universidade de São Paulo São Paulo Brazil; ^5^ School of Cancer & Pharmaceutical Sciences Faculty of Life Sciences & Medicine King's College London London UK; ^6^ Aulesa Biosciences Ltd Shefford UK; ^7^ Faculdade de Ciências Farmacêuticas Universidade de São Paulo São Paulo Brazil

**Keywords:** cnidaria, fitness, proteomics, reciprocal transplantation, toxin diversification, venom

## Abstract

*Tubastraea coccinea* is an azooxanthellate coral species recorded in the Indian and Atlantic oceans and is presently widespread in the southwestern Atlantic with an alien status for Brazil. *T. coccinea* outcompete other native coral species by using a varied repertoire of biological traits. For example, *T. coccinea* has evolved potent venom capable of immobilizing and digesting zooplankton prey. Diversification and modification of venom toxins can provide potential adaptive benefits to individual fitness, yet acquired alteration of venom composition in cnidarians is poorly understood as the adaptive flexibility affecting toxin composition in these ancient lineages has been largely ignored. We used quantitative high‐throughput proteomics to detect changes in toxin expression in clonal fragments of specimens collected and interchanged from two environmentally distinct and geographically separate study sites. Unexpectedly, despite global changes in protein expression, there were no changes in the composition and abundance of toxins from coral fragments recovered from either site, and following clonal transplantation between sites. There were also no apparent changes to the cnidome (cnidae) and gross skeletal or soft tissue morphologies of the specimens. These results suggest that the conserved toxin complexity of *T. coccinea* co‐evolved with innovation of the venom delivery system, and its morphological development and phenotypic expression are not modulated by habitat pressures over short periods of time. The adaptive response of the venom trait to specific predatory regimes, however, necessitates further consideration.

## Graphical abstract

Toxin complexity in the alien coral *Tubastraea coccinea* is conserved and co‐evolved with innovation of the venom delivery system. Morphological development of the coral and phenotypic expression of the venom and delivery system is not modulated by habitat pressures over short periods of time.

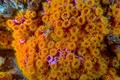

## 1 INTRODUCTION

The major and most lethal components of animal venoms are protein and peptide toxins. The toxin compositions of venom are attributed to serve in both offensive and defensive functions to facilitate prey capture and provide protection from predation (Casewell, Wüster, Vonk, Harrison, & Fry, 2013). Animals must adjust to divergent and changing conditions in their biotic and abiotic environment, which offer opportunities or pose challenges for feeding and defense. Adapting toxin composition to accommodate these changes has been extensively documented in prominent venomous bilaterians. For example, variability in snake venom toxins has been associated with geographical distribution (Alape‐Girón et al., 2008) and their ecological conditions (Strickland et al., 2018), but are most widely attributed with their ability to capture, consume, and digest a wide variety of different prey types (Daltry, Wüster, & Thorpe, 1996; Gibbs & Mackessy, 2009). Likewise, geographical variations in venom toxin composition have been documented in some scorpions, spiders, and species of cone snails, which has been linked also to changes in habitat or diet (Abdel‐Rahman, Omran, Abdel‐Nabi, Ueda, & McVean, 2009; Duda, Chang, Lewis, & Lee, 2009; Pekár, Petráková, Šedo, Korenko, & Zdráhal, 2018). However, data are scarce or nonexistent for geographical or intraspecific variation for the majority of the venomous taxa.

Cnidaria (corals, sea anemones, hydroids, jellyfish, myxozoans, and kin) possess a unique venom delivery system–nematocytes or “stinging cells,” that release their toxic payload by discharging a penetrative barb from an intracellular cnida organelle called a nematocyst. Approximately 30 different varieties of nematocyst are known to exist, but individual species usually combine no more than two to six structural types that are collectively known as the organism's cnidome (Östman, 2000). Cnidarians are possibly the earliest diverging venomous animal lineage to deploy venom for both predation and defense (Jaimes‐Becerra et al., 2017). Venom production and maintenance are therefore central to cnidarian existence and evolution, and increasingly more is known about the evolutionary history and phyletic distribution of cnidarian toxins. A pattern is emerging which suggests venoms with predominantly cytolytic and neurotoxic activities were established by early cnidarian ancestors followed by lineage‐specific recruitment of certain toxin protein families, with cytolysins diversifying prominently in Medusozoa and neurotoxins in Anthozoa (Balasubramanian et al., 2012; Brinkman et al., 2015; Huang et al., 2016; Jaimes‐Becerra et al., 2017; Li et al., 2014, 2016; Macrander, Brugler, & Daly, 2015; Madio, Undheim, & King, 2017; Ponce, Brinkman, Potriquet, & Mulvenna, 2016; Rachamim et al., 2015). When an innovative unsupervised clustering approach was used to compare toxin composition between groups of venomous animals, the results revealed that despite the early divergence and morphological simplicity of cnidarians, their toxin composition was as complex as those of venomous insects, gastropods, and elapid snakes (Jaimes‐Becerra et al., 2019).

**Figure 1:**
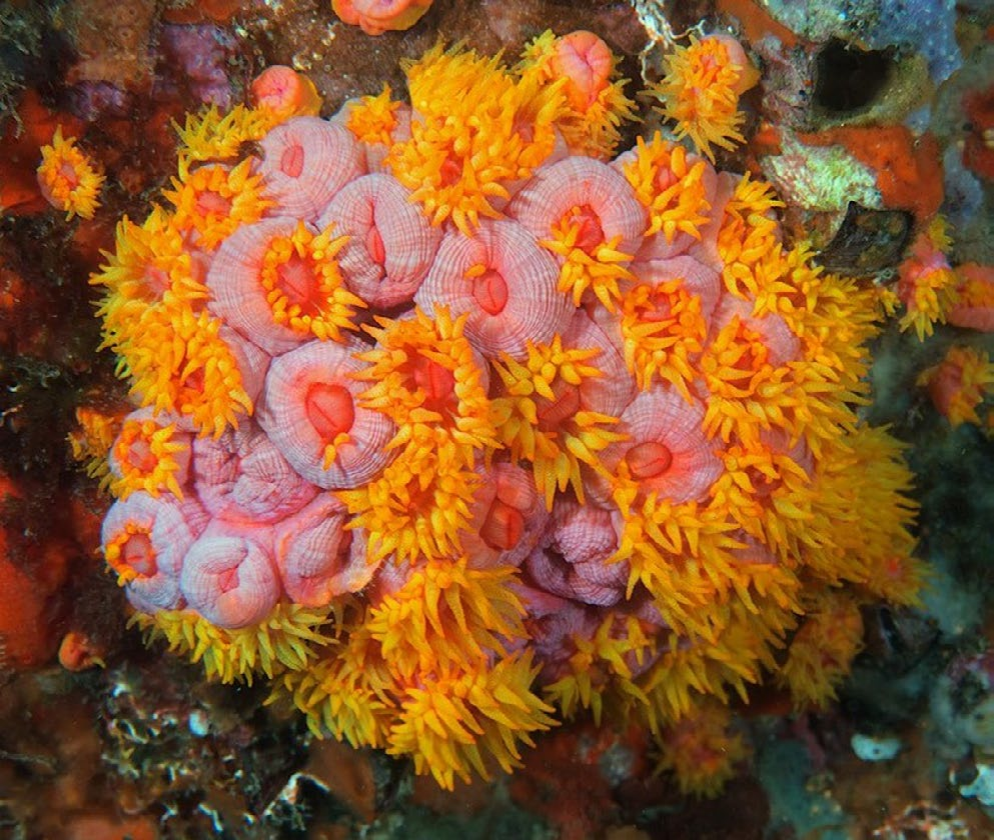
Gross skeletal and soft tissue morphology of *Tubastraea coccinea*

The orange cup coral *Tubastraea coccinea* Lesson 1829 (Figure 1) is presently broadly distributed in the Indian and Atlantic oceans. Corals of the genus *Tubastraea* are obligate heterotrophs, which lack autotrophic dinoflagellate endosymbionts to provide host photosynthetic nutritional energy, so instead feed predominantly on pelagic zooplankton. *T. coccinea* is considered an alien and invasive species to the southwestern Atlantic Ocean that has now expanded its habitat range to much of Brazil's southern coastal reefs, which is believed to have been propagated by a single invasion event in the 1990s (reviewed by Miranda, Costa, Lorders, Nunes, & Barros, 2016). *T. coccinea* is a hermaphroditic coral; therefore, settlement of broadcast planula via sexual reproduction does not occur. Proliferation ensues by the release of brooding larvae (Ayre & Resing, 1986) with high fecundity (de Paula, Pires, & Creed, 2014), or is spread by runners (Vermeij, 2005) and by the fragmentation of colonies, including regeneration from undifferentiated coral tissue which fosters the rapid propagation of clonal offspring (Capel, Migotto, Zilberberg, & Kitahara, 2014; Luz et al., 2018).

Recent proteomic analysis of nematocyst venom isolated from primary tentacles of *T. coccinea* revealed a complement of 17 likely toxins, mainly with predicted cytolytic or protease inhibitor activities, consistent with consuming a zooplankton diet (Jaimes‐Becerra et al., 2019). Given the vastly different trophic and interspecific interactions cnidarians encounter in diverse benthic and pelagic habitats, surprisingly little is known about their venom composition in response to diverse ecological conditions. Here, we use a tandem mass tag (TMT)‐based proteomics approach to compare wild clonal populations of *T. coccinea* from near‐shore and offshore habitats to determine whether geographical distribution and environmental factors could influence venom toxin modification in this early diverging metazoan, or whether such alterations are elaborated only by more advanced bilaterian taxa.

## 2 MATERIALS AND METHODS

### 2.1 Study sites and sampling design

**Figure 2:**
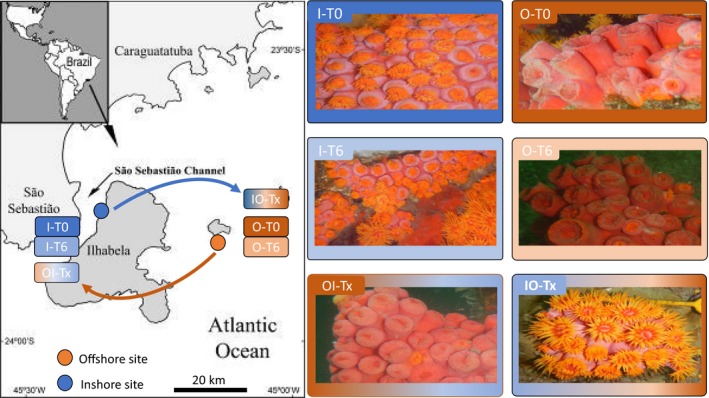
(a) Map of the study area and (b) morphology of *Tubastraea coccinea* samples during the study. (a) To assist orientation, the chart marks positions of the offshore and inshore experimental sites and depicts the direction of transplantations. (b) Photographs showing the gross morphology of coral fragments. Key: O, offshore site; I, inshore site; IO‐Tx, inshore to offshore transplant; OI‐Tx, offshore to inshore transplant; T0, start of the experiment; T6, end of experiment (6 weeks)

Clonal populations of *T. coccinea* from two environmentally distinct and geographically separate study sites were collected from the northern coast of São Paulo State, Brazil, and were analyzed for changes in protein expression at the beginning and end of a six‐week period of transplantation (Figure 2). All field work was conducted with appropriate permissions (MAA Research permit number 36717‐17). Reciprocal transplantations were made between an offshore site (Ilha dos Búzios; −23.81434, −45.20562) and an inshore site (Ilha Bella yacht club; −23.813611, −45.369699). The offshore and inshore sites are 40 km apart and separated by São Sebastião Island (Ilhabela municipality) and São Sebastião Channel. The offshore site has notably greater sea surface current speeds, lower temperature, and lower turbidity regimes compared to that of the inshore habitat site, but otherwise the two locations were similar in depth. In September 2017, large (approx.) 15 × 15 cm colonies of *T. coccinea* were collected from wild populations from each site and sectioned into nearly equal thirds. One third of each colony was replaced in its native habitat (designated offshore O‐T6 and inshore I‐T6), while a second fragment was transplanted to the alternate study site (Figure 2, designated offshore to inshore OI‐Tx and inshore to offshore IO‐Tx). The third fragment of each colony (designated offshore O‐T0 and inshore I‐T0) was taken to the Centro de Biologia Marinha (CEBIMar) and stored at −80°C. At completion of the field experiment, *T. coccinea* fragments from original and transplanted offshore and inshore sites were recovered and stored also at −80°C for subsequent proteomics analyses. The gross morphological features of each coral fragment were photographed at the beginning and end of the transplant experiment.

### 2.2 Isolation and identification of nematocysts

Individual *T. coccinea* polyps are relatively large, and although the tentacles retract upon freezing, the soft tentacle tissues could easily be dissected using forceps and a scalpel blade. Intact nematocysts were isolated from the dissected tentacles as previously described (Weston et al., 2013) and, after microscopic inspection, were lyophilized. An optical microscope (Nikon ECLIPSE 80i with a 100x objective lens with immersion oil) equipped with a digital camera (Nikon DS‐Ri1) for documentation was used for the identification of nematocysts. The abundance of different nematocyst types was recorded as either “very common,” “common,” or “rare” based on the scheme of Picciani, Pires, and Silva (2011).

### 2.3 Protein extraction

A volume of 200 µl of lysis buffer (8 M urea, 75 mM NaCl, 50 mM Tris, pH 8.2, plus one tablet each of Roche protease (cOmplete) and phosphatase (PhosStop) inhibitor cocktail per 10 ml of lysis buffer) was added to approximately 10 mg of freeze dried nematocyst tissue. The reconstituted material was disrupted on ice using a Qiagen TissueLyser II operated at 20–30 Hz for 2 min. The extracts were then centrifuged for 5 min at 10,000 × *g* and 4°C. The supernatants were decanted, and soluble protein concentrations were quantified by Nanodrop (Thermo Scientific) spectrophotometry and then lyophilized.

### 2.4 Protein preparation and labeling

The lyophilized samples were reconstituted in 50 µl TEAB and 1 µl of each protein extract (equivalent to 25 µg of protein in each sample), protein disulfides were reduced with 10 µl of 8 mM TCEP in 100 mM TEAB and 0.1% (w/v) SDS at 55°C for 1 hr, and then alkylated with 10 µl of 67.5 mM iodoacetamide in 100 mM TEAB and 0.1% (w/v) SDS at room temperature for 30 min in the dark. Trypsin digestion was then performed overnight at 37°C by adding 10 µl of 0.2 mg/ml trypsin in 100 mM TEAB and 0.1% (v/v) TFA. Following trypsin digestion, each sample was labeled with an isobaric tandem mass tag (TMT) 6‐plex reagent set using the protocol supplied with the reagent kit (Lot# SF251226 Thermo Fisher). Once labeled with a unique TMT reagent, the six individual samples were combined to create the TMT6plex analytical sample mixture. This combined sample of TMT labeled peptides was then fractionated into 12 fractions by off‐gel electrophoresis using an IPG strip pH 3–10 (Bio‐Rad) for 20 kV hr in 1× OFFGEL buffer, pH 3–10. Fractions were solubilized in 50 mM ammonium bicarbonate prior to LC‐MS/MS.

### 2.5 LC‐MS/MS tandem mass spectrometry

Chromatographic separations were performed on each fraction using an Ultimate 3000 nano‐LC system in line with an Orbitrap Fusion Tribrid mass spectrometer (Thermo Scientific). In brief, peptide samples in 1% (v/v) formic acid were injected onto an Acclaim PepMap C18 nano‐trap column (Thermo Scientific). After washing with 0.5% (v/v) acetonitrile and 0.1% (v/v) formic acid, peptides were resolved on a 250 mm × 75 μm Acclaim PepMap C18 reverse‐phase analytical column (Thermo Scientific) over a 150‐ min organic gradient, using 7 gradient segments (1%–6% solvent B over 1 min, 6%–15% B over 58 min, 15%–32% B over 58 min, 32%–40% B over 5 min, 40%–90% B over 1 min, held at 90% B for 6 min, and then reduced to 1% B over 1 min.) with a flow rate of 300 nl/min. Solvent A was 0.1% (v/v) formic acid, and Solvent B was aqueous 80% (v/v) acetonitrile in 0.1% (v/v) formic acid. Peptides were ionized by nano‐electrospray ionization at 2.2 kV using a stainless‐steel emitter with an internal diameter of 30 μm (Thermo Scientific) and a capillary temperature of 250°C. All spectra were acquired using an Orbitrap Fusion Tribrid mass spectrometer controlled by Xcalibur 2.0 software (Thermo Scientific) and operated in data‐dependent acquisition mode. FTMS1 spectra were collected at a resolution of 120,000 over a scan range (*m*/*z*) of 350–1,550, with an automatic gain control (AGC) target of 400,000 and a maximum injection time of 100 ms. The data‐dependent mode was set to cycle time with 3 s between master scans. Precursors were filtered according to charge state (to include charge states 2–7), with monoisotopic precursor selection and using an intensity range of 5E3 to 1E20. Previously interrogated precursors were excluded using a dynamic window (40 s ± 10 ppm). The MS2 precursors were isolated with a quadrupole mass filter set to a width of 1.6*m*/*z*. ITMS2 spectra were collected with an AGC target of 5,000, maximum injection time of 50 ms, and HCD collision energy of 35%.

### 2.6 Data analysis

PEAKS v8.5 software (Bioinformatics Solutions Inc.,) was used for de novo sequencing, annotation, and quantification of unique MS/MS events (File S1 in the PRIDE repository dataset identifier PXD015559). Potential toxin sequences were annotated by homology searching against a custom toxin database comprising the UniProtKB/SwissProt‐ToxProt dataset (Jungo, Bougueleret, Xenarios, & Poux, 2012) and previously published putative cnidarian toxin proteins (Jaimes‐Becerra et al., 2019). Protein sequences with an *e*‐value of <1.0e^–5^ to entries in the custom toxin database were used as input to a machine learning tool called “ToxClassifier” that excludes proteins with possible nontoxic physiological functions (Gacesa, Barlow, & Long, 2016). Sequences that met the stringent validation process were considered *bone fide* potential toxins. A second analysis was carried out to assign putative biological functions to sequences not identified as potential toxins, by comparison against the annotated transcriptome of *Eguchipsammia fistula* (Yum et al., 2017), the closest species to *T. coccinea* for which gene predictions are available. Sequences were assigned an annotation that gave a match against the *E. fistula* transcriptome with an *e*‐value of <1.0e^–5^. The PEAKS Q algorithm was used to measure the abundance of peptides across the TMT6plex dataset where TMT reporter ion signals were above the limit of quantitation (LOQ value). The value for each peptide in a protein was summed into one measurement according to an up(+) or down(−) fold‐change calculation. The quantitative results were represented as a clustered heat map (double dendrogram) using Neighbor‐joining tree clustering based on the Euclidean distance. MS/MS spectra corresponding to regulated features of interest were manually reviewed to validate the assignment, and these raw spectral data have been deposited via the PRIDE partner repository with the dataset identifier PXD015559.

## 3 RESULTS

There were no apparent gross morphological differences among the coral fragments collected from the offshore or inshore sites at T0 and T6; and the transplanted Tx fragments recovered at the two experimental sites. The coral skeleton and soft tissues formed typical clumps of pink‐red calcareous cups around a single deep red or orange polyp, with yellow to bright orange tentacles (Figure 2). The composition and abundance of cnidae types were also uniform between T0, T6, and Tx offshore and inshore fragments, consistent with expected variation between specimens (Table 1). The most common capsule types were spirocysts, tentacle type holotrichous and tentacle type b‐rabdoids; while mesentery type holotrichous and mesentery type b‐rabdoids were also noticed, but to a lesser extent. All morphological features are consistent for this species (Picciani et al., 2011).

**Table 1:** Cnidome composition of *Tubastraea* *coccinea* specimens sampled offshore, inshore and those transplanted between the two sites

Treatment	Offshore	Inshore
T0	Spirocyst—very common Tentacle type holotrichous—common Tentacle type b‐rhabdoid—common	Spirocyst—very common Tentacle type holotrichous—common Tentacle type b‐rhabdoid—common Mesentery type holotrichous—rare Mesentery type b‐rhabdoid—rare
T1	Spirocyst—very common Tentacle type holotrichous—common Tentacle type b‐rhabdoid—common Mesentery type holotrichous—rare Mesentery type b‐rhabdoid—rare	Spirocyst—very common Tentacle type holotrichous—common Tentacle type b‐rhabdoid—common
Tx	Spirocyst—very common Tentacle type holotrichous—common Tentacle type b‐rhabdoid—common Mesentery type holotrichous—rare Mesentery type b‐rhabdoid—rare	Spirocyst—very common Tentacle type holotrichous—common Tentacle type b‐rhabdoid—common

Note

The identification and abundance of different nematocyst types followed the scheme of Picciani et al. (2011).

The composition of recognized toxins extracted from specimens' nematocysts was identical irrespective of site, time of sampling, and transplantation (File S1 PRIDE repository dataset identifier PXD015559). The venom of *T. coccinea* is composed of 19 identified protein toxins with predicted neurotoxic (8/19), cytolytic (6/19), or dyshomeostasis (5/19) toxicological functions (Table 2). No significant effect of site or treatment was found on the fold‐change expression of the 10 putative toxins which met the criteria for quantification (proteins used for quantification are labeled in Table 2 and the quantification data are given in File S1(PRIDE repository dataset identifier PXD015559).

**Table 2:** Predicted venom proteome of potential toxins from nematocysts of *Tubastraea* *coccinea*

Toxin with closest homology	Possible toxin function	Accession numbers		Example of animal species with closest homology
α‐Latrotoxin‐Lhe1a	Neurotoxin^a^	Toxin3081	P0DJE3	*Latrodectus hesperus (Western black widow spider)*
Calglandulin	Neurotoxin^a^	Toxin6709	adi_v1.03437*	*Acropora digitifera* (Staghorn coral)
Calglandulin	Neurotoxin	Toxin6710	adi_v1.01102*	*Acropora digitifera* (Staghorn coral)
Basic phospholipase A2	Neurotoxin^a^	Toxin3317	O42187	*Gloydius halys (*Siberian pit viper*)*
Basic phospholipase A2	Neurotoxin	Toxin3579	P14556	*Naja pallida (Red spitting cobra)*
Ω‐theraphotoxin‐Hs1a	Neurotoxin	Toxin5919	P68424	*Haplopelma schmidti (Chinese bird spider)*
Stonustoxin subunit‐α	Neurotoxin	Toxin4963	Q98989	*Synanceia horrida* (Estuarine stonefish)
K^+^ channel toxin α‐KTx 4.2	Neurotoxin	Toxin2551	P56219	*Tityus serrulatus* (Brazilian yellow scorpion)
Endothelin‐converting enzyme 1	aCytolysin	Toxin6732	Ponce et al. (2016)	*Chrysaora* fuscescens (Pacific sea nettle)
Gigantoxin‐4	Cytolysin^a^	Toxin6744	H9CNF5	*Stichodactyla gigantea* (Giant carpet anemone)
Waprin‐Phi1	Cytolysin^a^	Toxin6649	A7X4K1	*Philodryas olfersii* (South American green snake)
Phospholipase D	Cytolysin^a^	Toxin0776	C0JB53	*Sicarius peruensis (Six‐eyed sand spider)*
Phospholipase D	Cytolysin	Toxin0293	C0JB21	*Loxosceles rufescens (Recluse spider)*
Phosphodiesterase	Cytolysin^a^	Toxin6676	adi_v1.12125*	*Acropora digitifera* (Staghorn coral)
Disintegrin	Dyshomeostasis^a^	Toxin6705	adi_v1.15751*	*Acropora digitifera* (Staghorn coral)
Disintegrin	Dyshomeostasis	Toxin6487	P18619	*Protobothrops flavoviridis (Habu pit viper)*
Disintegrin	Dyshomeostasis	Toxin6701	adi_v1.17845*	*Acropora digitifera* (Staghorn coral)
Snaclec 7	Dyshomeostasis	Toxin6686	adi_v1.12298*	*Acropora digitifera* (Staghorn coral)
Flavoxobin	Dyshomeostasis^a^	Toxin4271	P05620	*Protobothrops flavoviridis (Habu pit viper)*

Note

Putative toxins were annotated by homology of peptide sequences obtained from de novo sequencing of unique peptide MS/MS events with a custom database of known animal venom toxins. Venomous animals and their toxins with closest sequence similarity are given together with accession numbers corresponding to either UniProt or * ZoophyteBase (Dunlap et al., 2013) assignments. Note that the accession numbers in the left‐hand column refer to the laboratory numbers used for the proteomics analysis given in File S1 (PRIDE repository dataset identifier PXD015559).

a Potential toxins which met the criteria for quantification, data and calculations for which are given in File S1 (PRIDE repository with the dataset identifier PXD015559).

**Figure 3:**
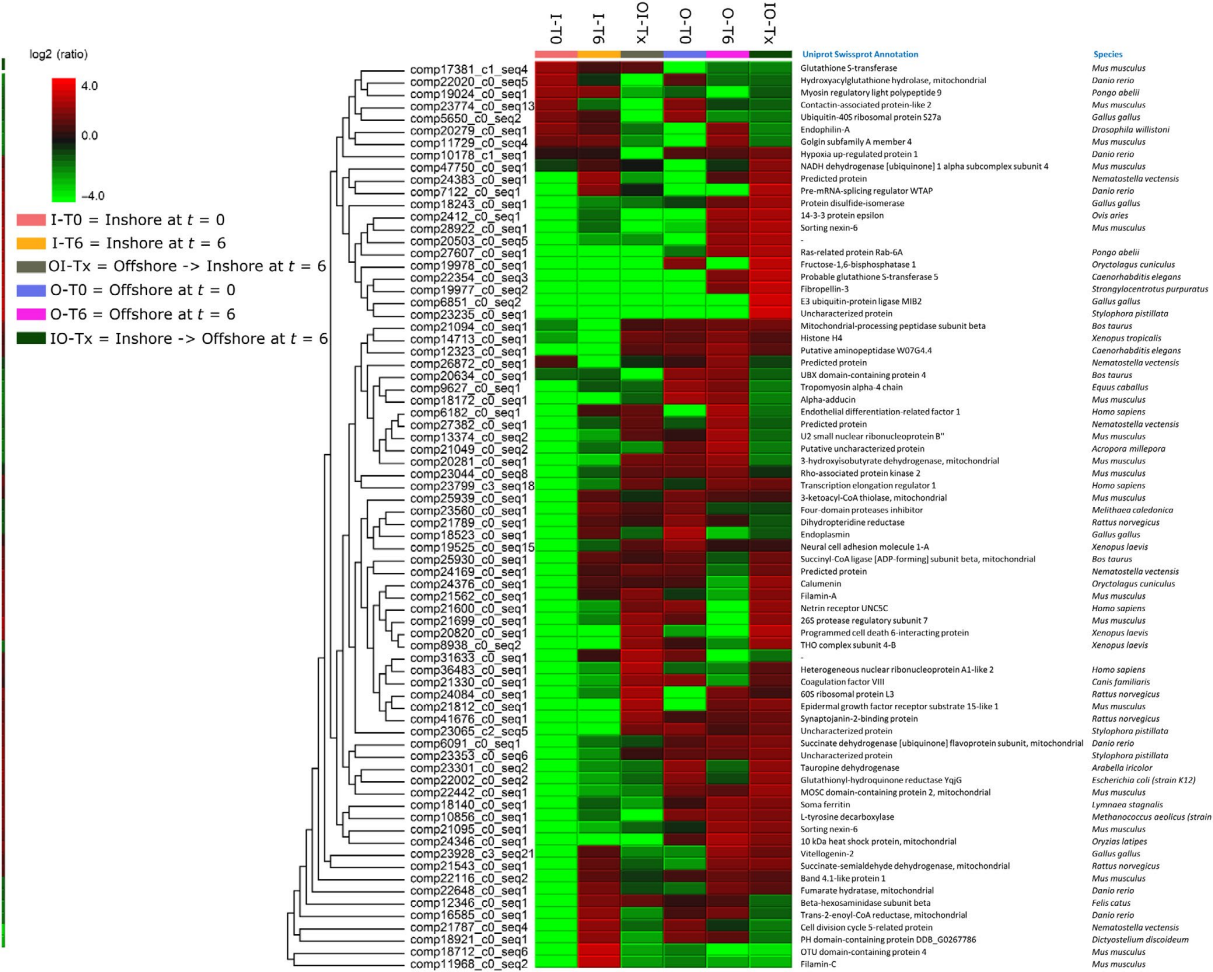
Heatmap showing quantified changes of proteins from discharged nematocysts isolated from *Tubastraea coccinea* specimens sampled offshore, inshore, and those transplanted between the two sites. Fold‐change values were considered either a significant reduction (<2) or significant increase (>2) in tag intensity

An additional 74 proteins from the discharged nematocysts were annotated by molecular comparison to the published *E. fistula* transcriptome (Yum et al., 2017); these nontoxin proteins were common in all samples and met the criterion for quantification (File S2 PRIDE repository dataset identifier PXD015559). The average fold‐change of peptides associated with each site and treatment is displayed as a heat map (Figure 3), in which values <2 or >2 were considered either a significant reduction or increase in protein tag intensity. Overall, the trends in nontoxin peptide fold‐changes were different at T0 and T6 (i.e., six weeks) between offshore and inshore coral fragments (File S2 PRIDE repository dataset identifier PXD015559). Corals at the offshore site shared 50/74 (67.6%) of proteins with the same levels of expression between the T0 and T6 specimens. Likewise, 41/74 (55.4%) of proteins did not have significant differences in expression between the T0 and T6 specimens at the inshore site. Hence, fold‐change comparisons were made between the T6 and Tx proteomes (i.e., at six weeks only). Transplantation between sites had a marked effect on protein fold‐changes (Figure 3 and File S2 PRIDE repository dataset identifier PXD015559). After six weeks, the Tx fragment transplanted from the offshore to inshore site retained 13/74 (17.6%) of proteins with similar levels of expression to corresponding proteins in the native offshore T6 proteome. The fold‐changes in 30/74 (40.5%) of proteins in this Tx specimen significantly shifted six weeks after transplantation to resemble levels of expression to the same proteins in the T6 coral at the inshore site. Surprisingly, 31/74 (41.9%) of the Tx proteome had protein fold‐changes that were significantly different to the T6 specimen from the offshore to inshore sites. Protein fold‐changes in the nontoxin proteome of the Tx fragment relocated from inshore to offshore sites was also affected six weeks after transplantation. While fold‐changes in 20/74 (27.0%) of proteins were expressed at identical levels as those of the T6 specimen at the inshore site, 24/74 (32.4%) of proteins shifted to levels of expression equivalent to the native T6 fragments collected at the offshore site. For 30/74 (40.5%) of proteins in this Tx fragment, fold‐changes did not resemble the levels of protein expression in either of the local T6 coral fragments from offshore or inshore sites.

## 4 DISCUSSION

This study examined potential differences in venom toxins of *T. coccinea* colonies inhabiting an inshore and offshore site in southern Brazil. The potential for *T. coccinea* venom to adapt its toxin composition in response to different habitat environments was also assessed by manipulating a 6‐week reciprocal transplant trial between the study sites. While there were some significant site‐specific expression differences in the global proteome of *T. coccinea* and its response to reciprocal transplantation over the 6‐week study period (Figure 3 and File S2 PRIDE repository dataset identifier PXD015559), quantitative proteomic analyses clearly show that there was no difference in venom toxin expression in populations inhabiting the geographical confines of our study area. These results are surprising because animal venoms are typically composed of many constituent toxins that together manifest as the venom phenotype. Hence, we can hypothesize that strong selection is acting upon these toxic constituents to refrain adaptation to possible differences in feeding and defense pressures across geographically and environmentally separate habitats, although not necessarily distinct *T. coccinea* populations.

Dichotomy in venom phenotypes has been extensively described on geographical and environmental scales in many venomous bilaterians. These patterns were initially deduced upon observing dissimilar symptoms following envenomation occurring from a common species. More recently, the hypothesis was tested by direct toxin analysis and from next‐generation sequencing data annotation (Zancolli et al., 2019). A classic example of venom polymorphism in geographically distinct populations is the presence or absence of neurotoxic phospholipase A_2_ in Mojave Rattlesnakes (*Crotalus scutulatus*) across the Sonoran Desert of the southwestern United States and northern Mexico. The absence of the phospholipase confers a less potent hemorrhagic venom phenotype, resulting in local and differential selection for a greater proportion of mammals over lizard prey items (Strickland et al., 2018). A previous study that compared the toxicological characteristics of cnidarians, however, concurred with our results. In that study (Radwan et al., 2001), the cnidome toxin composition of *Cassiopea* and *Aurelia* species collected from very distinct habitats and geographical locations (viz. Bahamas, Chesapeake Bay, and Red Sea) were identical. The toxicological profiles of the venoms from isolated nematocysts were also alike between locations for each species; nevertheless, the potency of the venom in the Red Sea specimens was much greater (Radwan et al., 2001).

After six weeks, fragments of native and transplanted colonies collected at the sample sites retained identical macro (skeletal and soft tissue features, Figure 2) and micro (cnidome, Table 1) morphologies, as well as venom compositions (Table 2). The putative toxin proteome obtained from isolated tentacle nematocysts of *T. coccinea* was recently reported for specimens collected from the São Sebastião Channel in 2016 (Jaimes‐Becerra et al., 2019). The venom proteome of the 2016 specimen was also remarkably consistent with our findings presented herein, both in the number of constituent toxins and their predicted toxicological activities: 8/16 neurotoxic, 2/16 cytolytic and 6/16 dyshomeostatic toxins. Indeed, a similar observation was reached by comparing the venom proteomes of *Olindias sambaquiensis* from specimens collected 5 years apart attesting that cnidarian venom composition is stable over time, at least in populations of *O. sambaquiensis* from the São Sebastião Channel (Doonan et al., 2019). We are currently undertaking a 3‐year noninterrupted time‐series experiment to monitor the venom toxin composition of certain cnidarians in the São Sebastião Channel.

A recent study presented quantitative proteomics that accurately determined the composition and relative abundance of toxins present in enriched preparations of two nematocyst types isolated from the primary tentacles of the adult medusa stage of the hydrozoan *O. sambaquiensis*. The venom composition of the two capsule types was nearly identical, and there was little difference also in the relative abundance of toxins between the two nematocyst preparations (Doonan et al., 2019). A pattern is emerging from published data (Doonan et al., 2019; Radwan et al., 2001) and that of this study, whereby cnidarians appear to retain an array of different nematocyst types designed to deliver a single and invariant venom, with both venom and the delivery system having co‐evolved independently of geographical or environmental forces. Such may reveal, however, that relative concentrations of certain toxins may vary between nematocyst types (Doonan et al., 2019) or in species from different habitats, perhaps altering venom potency by varying nematocyst types and densities to allow adaptation concerning to prey preference or predator type, which warrants further investigation.

It may be argued that the six‐week study period was not long enough for *T. coccinea* to adapt change in its venom composition, cnidome, and gross morphology following transplantation between the offshore and inshore sites. *T. coccinea* is an azooxanthellate and obligate heterotroph coral with an extreme regenerative capacity (Luz et al., 2018) that depends on prey capture to meet its metabolic needs, including its reproductive energy demands. It was thus expected that the venom composition of *T. coccinea* would be different in colonies sampled at inshore and offshore sites, due to potential disparities in habitat prey populations. Yet, there were significant fold‐changes in the cnida proteomes of specimens collected at each site and those following reciprocal transplantation after six weeks of acclimation. Nevertheless, it should be noted that the natural distributional range of this southwestern Atlantic invasive species has a much more diverse coral fauna and, therefore, complex “within order” substrate competition. Such competition might have shaped *T. coccinea* venon/toxins, which are potent enough to outcompete the native species from invaded areas.

However, comparatively, it is evident that a six‐week period was sufficient for transcription and translation processes to alter expression patterns in the nontoxin proteome of *T. coccinea* nematocytes, but such adaptation certainly did not extend to the venom phenotype. Venomous anthozoans often elaborate the same combination and potency of toxins for both predation and defense, potentially where constitutive expression is adequate for both purposes, even during a severe “bleaching” event causing an enhance requirement for heterotrophic metabolism (Hoepner, Abbott, & Burke da Silva, 2019). Changes in predator exposure, but not diet, elicit a larger venom proportion of defensive toxins than predatory toxins in the scorpion *Liocheles waigiensis* (Gangur et al., 2017), and remarkably, carnivorous cone snails are reported to switch between distinct venoms in response to predatory or defensive stimuli (Duterte et al., 2014). The only known predators of *T. coccinea* in the tropical Indo‐Pacific are the gastropod *Epidendrium billeeanum* (Rodríguez‐Villalobos, Ayala‐Bocos, & Hernandez, 2016) and the nudibranch *Phyllida melanobrachia* (Okuda, Klein, Kinnel, Li, & Scheuer, 1982), while generalist predators are yet to be detected within invaded habitats of the southwestern Atlantic (Moreira & Creed, 2012). We are using time lapse photography in current studies to record predation of *T. coccinea* by native carnivores within our Brazilian study sites with a view to test the venom response of *T. coccinea* on exposure to sustained threats of predation.

Although the offshore and inshore sites were spatially separated and environmentally distinct habitats, it was assumed that the proximate composition of the pelagic community would be similar at both locations since epifaunal benthic populations were somehow alike. Thus, the venom phenotype would be unchanged if predation alone were selective and if abiotic factors were not driving toxin diversification, as well as if the potency of the venom toxins is adequate across a broad spectrum of ecotypes. Changes in diet have been associated with divergence in venoms during the early life history of cnidarians. For example, Underwood and Seymour (2007) have documented distinct differences in tentacle venom protein profiles using 1D SDS–PAGE analyses between juvenile and mature *Carukia barnesi* medusae (the highly toxic Australian Irukandji jellyfish). Likewise, Columbus‐Shenkar et al. (2018) found also that venom composition changed dramatically between early developmental stages of the starlet sea anemone, *Nematostella vectensis*. Our research will further test the influence of how diet affects the expressed venome composition, by selective transcription and translation of component toxins, which will improve our understanding on how venom evolved in early cnidarian development.

## 5 CONCLUSIONS

The complexity of toxins in isolated nematocysts from the heterotrophic coral *T. coccinea* did not vary significantly in genotypically identical colonies taken from offshore and inshore sites, or following reciprocal transplantation of clonal fragments between the two sites. The structural morphology and cnidome toxin composition also did not change during the 6‐week study period. These findings suggest that *T. coccinea* produces a single and relatively invariant venom, and that the venom and its delivery system are conservative and expressed independently of geographical influence and environmental selection in the invaded habitats of the northern São Paulo coast.

## CONFLICT OF INTERESTS

None declared.

## AUTHOR CONTRIBUTIONS

MVK and PFL designed research; MVK, AJB, EGM, MW, and PFL performed research; MW, GP, ACMa, and ACMo contributed analytical tools; and all authors analyzed data and wrote the paper.

## Data Availability

The mass spectrometry proteomics data have been deposited to the ProteomeXchange Consortium via the PRIDE (Perez‐Riverol et al., 2018) partner repository with the dataset identifier PXD015559.
